# Structural dynamics, pathogenic mechanisms, and therapeutic targeting of the NLRP3 inflammasome in lupus nephritis

**DOI:** 10.3389/fimmu.2026.1904216

**Published:** 2026-08-24

**Authors:** Hongfei Sun, Xinran Xie, Mingru Ma, Hongbin Li, Yong Jin, Manling Zhang

**Affiliations:** 1 Department of Rheumatology and Immunology, The Affiliated Hospital of Inner Mongolia Medical University, Hohhot, China; 2 Inner Mongolia Key Laboratory for Pathogenesis and Diagnosis of Rheumatic and Autoimmune Diseases, The Affiliated Hospital of Inner Mongolia Medical University, Hohhot, China; 3 School of Basic Medicine Sciences, Inner Mongolia Medical University, Hohhot, China; 4 The State Key Laboratory of Reproductive Regulation and Breeding of Grassland Livestock, College of Life Science, Inner Mongolia University, Hohhot, China

**Keywords:** lupus nephritis, NLRP3 inflammasome, signaling pathways, structural characteristics, targeted therapy

## Abstract

Lupus nephritis (LN) is a severe complication of systemic lupus erythematosus (SLE). The nucleotide-binding oligomerization domain-like receptor family pyrin domain-containing 3 (NLRP3) inflammasome plays a central role in the pathogenesis of LN. Aberrant activation of the NLRP3 inflammasome directly promotes glomerular injury, proteinuria, and tubulointerstitial fibrosis through the induction of pyroptosis and the release of interleukin-1β (IL-1β) and IL-18. The expression level of the NLRP3 inflammasome is positively correlated with disease activity, making it both a key driver of acute renal injury in LN and a promising therapeutic target. In this review, a comprehensive summary of the structural features of NLRP3 is provided, including the pyrin domain (PYD), the nucleotide-binding oligomerization domain (NACHT), and the leucine-rich repeat domain (LRR). Additionally, the interaction between NIMA-related kinase 7 (NEK7) and the LRR domain is characterized, as well as the role of the fish-specific NACHT-associated (FISNA) domain in oligomerization. The canonical and non-canonical activation signaling pathways of the NLRP3 inflammasome are thoroughly analyzed. The pathological roles of the NLRP3 inflammasome in kidney-resident cells, including podocytes and renal tubular epithelial cells, as well as infiltrating immune cells, such as macrophages and T cells, are comprehensively discussed. Its crosstalk with multiple signaling pathways is also examined. Finally, the therapeutic potential of various intervention strategies, including direct inhibitors targeting NLRP3, upstream signaling pathway modulators, natural compounds, and novel gas therapies, in preclinical models of LN is evaluated. This review provides a comprehensive overview of the role of the NLRP3 inflammasome in LN and offers a theoretical basis for the development of novel NLRP3-targeted therapeutic strategies.

## 1 Introduction

Systemic lupus erythematosus (SLE) is a prototypical autoimmune disease characterized by the production of autoantibodies and multisystem involvement (1). Lupus nephritis (LN) is considered one of the most severe complications of SLE, affecting approximately 25%–60% of patients and occurring predominantly within the first 5 years after disease onset (2). The incidence and prevalence of LN are influenced by a multitude of factors, including age, sex, ethnicity, and socioeconomic status (3). Despite the continuous advancement in treatment, LN persists as a significant cause of mortality in patients with SLE. Approximately 5%–30% of patients progress to end-stage renal disease within 10 years of diagnosis, which is itself among the leading causes of death in SLE (4, 5). At present, glucocorticoids in combination with various immunosuppressive agents remain the cornerstone of LN treatment (6). Recent studies have demonstrated that the incorporation of obinutuzumab into conventional therapeutic regimens can enhance treatment efficacy (7). Despite the substantial advancements in disease control and patient survival, LN persists as a pivotal contributor to the long-term prognosis of patients with SLE (8).

The pathogenesis of LN is a highly intricate process. Traditionally, LN has been regarded as an immune complex (IC)-mediated disease, characterized by the deposition of ICs formed by double-stranded DNA and anti-double-stranded DNA antibodies (dsDNA–anti-dsDNA ICs) within the subendothelial and/or subepithelial regions of the glomerular basement membrane. This deposition activates the complement cascade, thereby initiating inflammatory responses. Activated complement components act as potent chemoattractants, recruiting both innate and adaptive immune cells to the kidney and thereby promoting inflammatory tissue injury (9, 10). However, emerging evidence indicates that, in addition to infiltrating immune cells, kidney-resident cells actively contribute to renal inflammation and immune responses. These cells, including glomerular mesangial cells (GMCs), podocytes, intrarenal macrophages, renal tubular epithelial cells (RTECs), and endothelial cells, are not only the targets of inflammatory injury but also important regulators of the local immune microenvironment (11).

The innate immune response constitutes the primary line of defense against injury and is initiated through the activation of pattern recognition receptors (PRRs) located either on the cell surface or within inflammatory cells (12). PRRs recognize pathogen-associated molecular patterns (PAMPs), damage-associated molecular patterns (DAMPs), and other danger signals. Upon activation, these cells promote the maturation and release of the pro-inflammatory cytokines interleukin-1β (IL-1β) and IL-18, thereby initiating downstream inflammatory responses and pyroptosis. PRRs are broadly classified into membrane-bound and intracellular receptors. Membrane-bound PRRs recognize extracellular ligands, whereas intracellular PRRs detect diverse cytoplasmic danger signals and interact with multiple adaptor proteins to form signaling complexes that activate caspase-1. Activated caspase-1 subsequently cleaves the inactive cytokine precursors pro-IL-1β and pro-IL-18 into their biologically active forms (13, 14). Among the intracellular PRRs, nucleotide-binding oligomerization domain-like receptor family pyrin domain-containing 3 (NLRP3) is known to be activated by a wide range of stimuli, including infection, tissue injury, and metabolic disturbances. Upon activation, NLRP3 oligomerizes with apoptosis-associated speck-like protein containing a caspase recruitment domain (CARD) (ASC) to form the NLRP3 inflammasome. This complex activates caspase-1, resulting in the maturation and secretion of IL-1β and IL-18 and the amplification of inflammatory responses (15).

In recent years, the critical role of the NLRP3 inflammasome in the pathogenesis of LN has attracted considerable attention. Research has indicated a positive correlation between NLRP3 expression and the SLE Disease Activity Index (SLEDAI) as well as renal histopathological activity, suggesting that NLRP3 is actively involved in the acute inflammatory processes underlying the disease (16). Moreover, experimental studies have shown that inhibition of NLRP3 activation effectively attenuates renal inflammation, ameliorates histopathological injury, and reduces proteinuria, underscoring the potential of NLRP3 as a promising therapeutic target for LN (17). In consideration of the aforementioned evidence, this review provides a comprehensive overview of recent advances in the understanding of NLRP3 structure, its interaction domains, and the signaling pathways governing NLRP3 inflammasome activation. It further examines the role of the NLRP3 inflammasome in the initiation and progression of LN and discusses the therapeutic potential of targeting NLRP3, with particular emphasis on current inhibitory strategies and their underlying mechanisms. By synthesizing the latest evidence, our objective is to provide a theoretical framework for the development of novel NLRP3-targeted therapies for LN.

## 2 Composition of the NLRP3 protein and recent discoveries of its interaction domains

**Figure 1:**
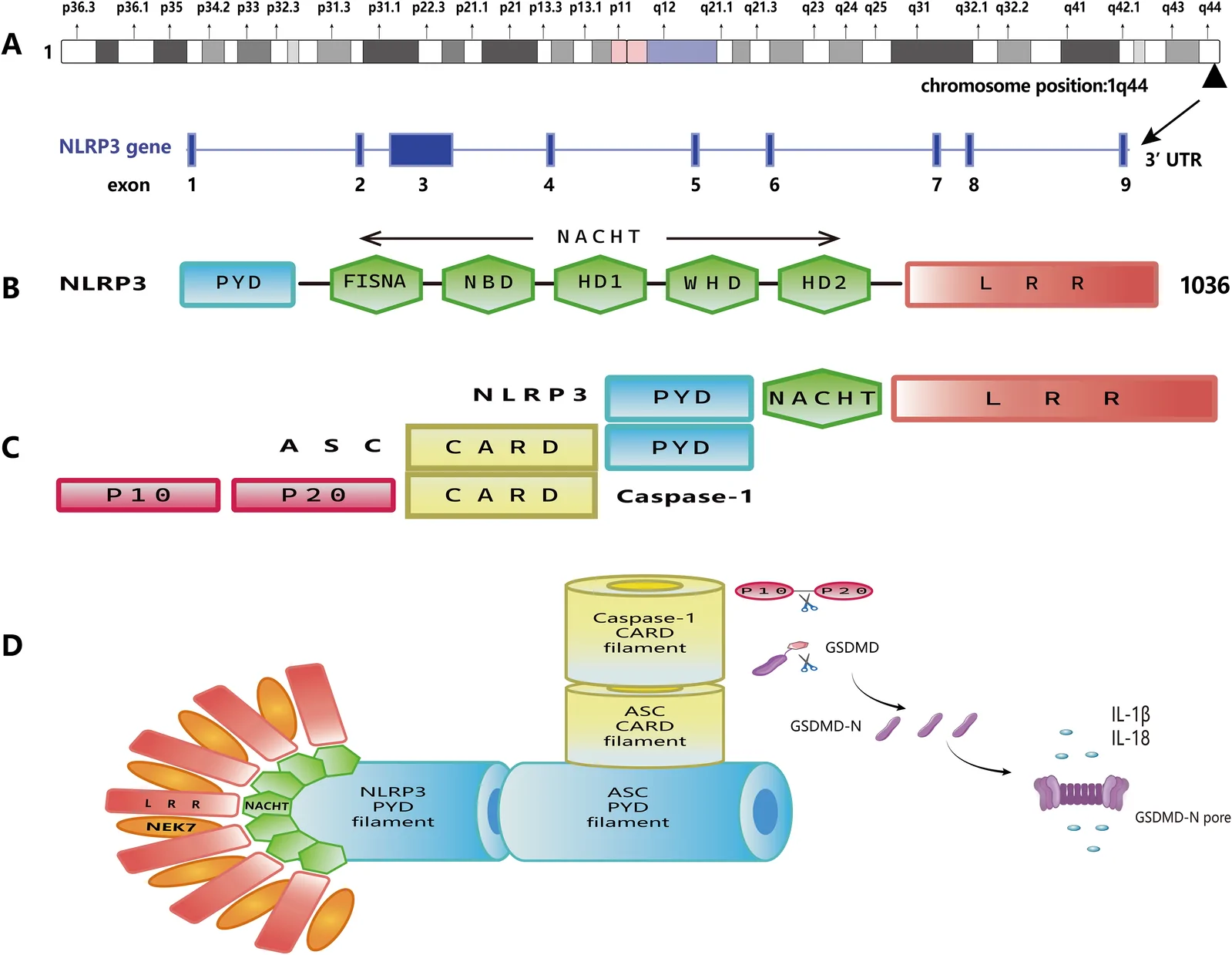
Gene structure, protein domains, and assembly of the NLRP3 inflammasome. **(A)** Structure of the human NLRP3 gene. The NLRP3 gene is located on chromosome 1 q44 and comprises nine coding exons. **(B)** Composition of the NLRP3 protein domain. **(C)** Sensor–adaptor–effector architecture of the NLRP3 inflammasome, illustrating the interaction of NLRP3 with ASC through their PYDs, and the interaction of ASC with caspase-1 through their CARDs. **(D)** A molecular model of the NLRP3 inflammasome pathway is presented, from upstream to downstream. Conformational changes in NLRP3 and its subsequent intracellular trafficking result in the binding of NLRP3 to NEK7, which induces the formation of the NLRP3 inflammasome disk structure and promotes the formation of a filament by the NLRP3 PYD. The NLRP3 PYD filament recruits ASC by nucleating the ASC PYD filament. The CARD of ASC also converges to form a filament. The ASC CARD filament recruits caspase-1 by nucleating the caspase-1 CARD filament. The caspase domain (p20/p10) of caspase-1 dimerizes and undergoes self-processing, thereby generating active caspase-1. Active caspase-1 subsequently cleaves pro-cytokines to generate mature cytokines. Caspase-1 also cleaves GSDMD to generate an active N-terminal fragment of GSDMD for membrane pore formation, which promotes cytokine release and pyroptosis (39).

Nod-like receptors (NLRs) comprise a large family of intracellular PRRs, whose members exhibit highly modular gene and protein structures (18). The human genome contains over 20 NLR genes, all of which share a conserved three-domain structural organization at both the gene and protein levels (19). The genetic composition of NLRP3 conforms to the core pattern of the family while also possessing unique structural features. The NLRP3 gene is located on chromosome 1q44 and spans approximately 25–31 kb. The gene consists of nine exons and encodes a protein consisting of 1,036 amino acids (20, 21) (**Figures 1A, B**). The promoter region of this gene contains binding sites for critical transcription factors such as NF-κB, and its expression is strictly regulated at the transcriptional level (22). Furthermore, mutations within the coding region of NLRP3 are associated with susceptibility to a wide range of chronic inflammatory diseases (23).

### 2.1 Fundamental structure of the NLRP3 protein

NLR family pyrin domain-containing protein 3 (NLRP3) is a key intracellular sensor that plays a critical role in mediating inflammasome formation. The domain composition of the protein dictates its recognition of danger signals, maintenance of an inactive state, and assembly into an activated inflammasome complex (24). The NLRP3 protein is composed of three essential domains: an N-terminal pyrin domain (PYD), a central nucleotide-binding oligomerization domain (NACHT), and a C-terminal leucine-rich repeat (LRR) domain (25) (**Figure 1B**).

**Figure 2:**
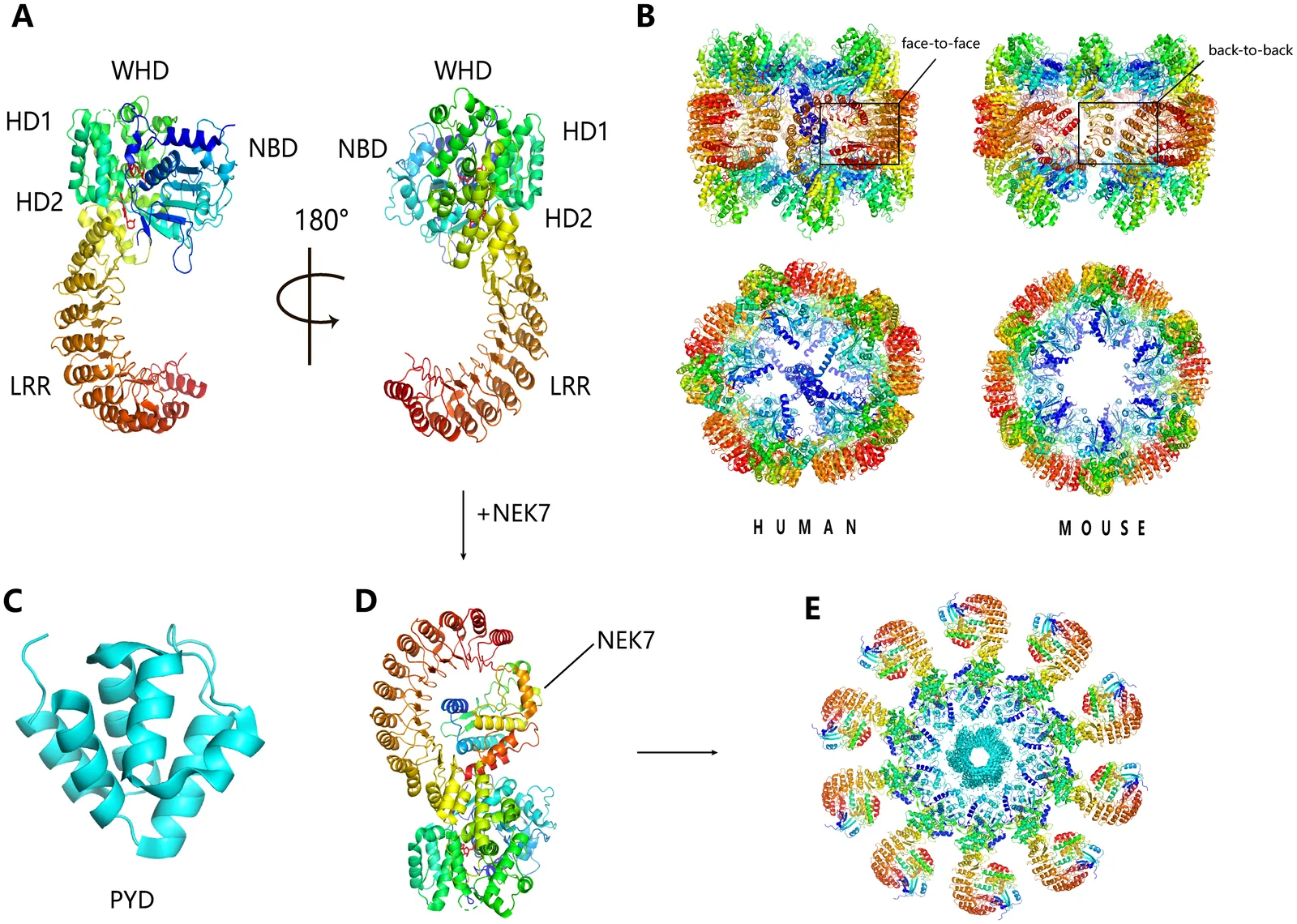
Structural organization and activation of NLRP3. **(A)** Ribbon diagram of PYD-deleted human NLRP3 (PDB: 9MIE). **(B)** Ribbon diagrams of the human full-length NLRP3 decamer (PDB: 7PZC) and the mouse full-length NLRP3 dodecamer (PDB: 7LFH), showing the “face-to-face” and “back-to-back” conformations (39). **(C)** Ribbon diagram of NLRP3 PYD (PDB: 3QF2). **(D)** Ribbon diagram of the NLRP3–NEK7 complex (PDB: 6NPY). **(E)** Top view of the active NLRP3 inflammasome disc structure (PDB: 8EJ4).

The PYD is located at the N-terminus of the NLRP3 protein and primarily functions as a mediator of protein–protein interactions. It functions as a dynamic regulatory module. Under resting conditions, PYD activity is restrained and maintained in a non-oligomerized state (**Figure 2C**). Upon stimulation, the overall conformation of NLRP3 changes, accompanied by dephosphorylation of the PYD. The dephosphorylated PYD then assembles into functional homofilaments that serve as nucleation platforms for the recruitment of ASC, thereby initiating inflammasome signaling (26).

Subsequent to NLRP3 activation, the exposed PYD recruits ASC through PYD–PYD homotypic interactions. ASC contains an N-terminal PYD and CARD. These proteins are recruited to oligomerized NLRP3 molecules via PYD–PYD homotypic interactions. The C-terminal CARD of ASC functions as a platform for the recruitment of effector caspase-1. Caspase-1 is composed of an N-terminal CARD, a C-terminal large catalytic subunit (p20), and a small catalytic subunit (p10). Interaction between the CARD domains of ASC and caspase-1 has been demonstrated to promote the formation of nucleated filaments, thereby facilitating the autocatalytic cleavage of caspase-1 into its active p20 and p10 subunits (24). Subsequent to this activation, the caspase-1 initiates the cleavage of a specific protein, gasdermin D (GSDMD). This process results in the release of a critical pore-forming fragment GSDMD-N, which rapidly traverses the cell membrane and integrates into its structure. The oligomerization of GSDMD-N results in the formation of large, non-selective, permeable pores on the membrane. This process enables the release of the mature inflammatory cytokines (IL-1β and IL-18) into the extracellular space. These cytokines recruit and activate additional immune cells, thereby amplifying the inflammatory response and contributing to the regulation of innate immune responses (27) (**Figures 1C, D**).

The NACHT domain constitutes the core component of the NLRP3 protein, playing a pivotal role in its oligomerization, ATPase activity, and inflammasome activation. Structurally, the NACHT domain is not a single unit but consists of five key subdomains: the fish-specific NACHT-associated (FISNA) domain, the nucleotide-binding domain (NBD), the helical domain 1 (HD1), the wing-shaped helical domain (WHD), and the helical domain 2 (HD2) (28) (**Figure 1B**). The primary function of the NACHT domain is to bind and hydrolyze ATP. In the inactive state, NLRP3 is predominantly ADP-bound. Upon activation, ADP is exchanged for ATP, resulting in a substantial conformational change within the NACHT domain. This ATPase activity is imperative for NLRP3 activation (29).

The LRR domain is located at the C-terminus of the NLRP3 protein and plays diverse and critical roles in the activation and regulation of the NLRP3 inflammasome. Nevertheless, its precise functions and mechanisms remain the focus of debate (30). It has been demonstrated that the replacement of the LRR domain with the LacZ protein, or the direct deletion of this domain, results in the elimination of NLRP3 inflammasome activation (31, 32). The LRR domain is composed of 11 LRR motifs, with a high proportion of leucine and cysteine residues, forming a characteristic horseshoe-shaped three-dimensional structure. Some of the cysteine residues are capable of sensing reactive oxygen species (ROS) (**Figure 2A**). Cryo-electron microscopy studies have revealed that, in the resting state, the LRR mediates the formation of a double-ring cage structure of NLRP3 through “back-to-back” and “face-to-face” interactions. Full-length mouse NLRP3 assembles into a 12-16-mer complex, whereas human NLRP3 adopts a decamer under resting conditions (24, 33) (**Figure 2B**). Conversely, other studies have suggested that the LRR domain is dispensable for NLRP3 inflammasome activation (34, 35).

### 2.2 Novel insights into the interaction between NEK7 and the LRR domain

In recent years, significant advancements have been made in elucidating the interaction between NIMA-related kinase 7 (NEK7) and the LRR domain of NLRP3, highlighting its pivotal role in regulating NLRP3 inflammasome activation (36). These findings have advanced our understanding of innate immune regulation and identified promising therapeutic targets for a broad spectrum of inflammatory diseases. NEK7 directly binds to the LRR domain of NLRP3, an interaction that is crucial for the oligomerization and final assembly of the inflammasome complex (30, 37) (**Figures 2D, E**).

It is noteworthy that the concave surface of the NLRP3 LRR domain functions not only as the binding site for NEK7 but also as the binding site for NLRP3 self-assembly within the caged structure. Consequently, when NEK7 binds to LRR, steric hindrance reduces the number of NLRP3 caged structures, resulting in smaller NLRP3 aggregates. This finding indicates that NEK7 disrupts the caged structure by competing with the NLRP3−NLRP3 binding site. The released NLRP3 is hypothesized to consist of two half-cages, which undergo conformational changes in the presence of ATP to reassemble into an active disc structure (38). Furthermore, NEK7 shares a partial binding site with the mitotic protein NEK9, which explains the mutual inhibition between NLRP3 activation and cell mitosis (39). The NEK7–LRR interaction plays a critical role in NLRP3 inflammasome activation, and the disruption of this protein–protein interface has emerged as a promising novel strategy for the treatment of NLRP3-associated diseases (40).

### 2.3 Role of the FISNA domain in NLRP3 assembly

The FISNA domain located within the NACHT domain of NLRP3 is a critical component of the NLRP3 protein. From the perspective of species comparison, the FISNA domain is present in NLRP3 proteins of various organisms, including humans and Atlantic salmon, thereby highlighting its functional importance. This domain contains an intrinsically disordered region, a characteristic that is essential for mediating the weak multivalent interactions required for NLRP3 assembly and activation (41).

A pivotal event in the conversion of NLRP3 from its ADP-bound inactive state to the active ATP-bound state is an approximate 85° rotation within the NACHT domain. This conformational change involves a relatively stable FISNA–NBD–HD1 module and the overall rotation of the WHD–HD2–LRR module. During this process, the newly formed Loop 1 and Helix 2 play essential roles in stabilizing the ATP-bound conformation and mediating interactions between adjacent NLRP3 subunits within the disc-shaped inflammasome. Preliminary experimental findings have demonstrated that mutations disrupting these interfaces markedly impair NLRP3 activation (38).

During oligomerization, the FISNA domain establishes a continuous interaction network between adjacent NLRP3 subunits through FISNA–FISNA and FISNA–NACHT interface interactions, thereby stabilizing the active-state oligomeric structure. Specifically, FISNA loop 1 in one subunit interacts with the “crocodile clip”-like structure formed by the NBD helix–loop–strand motif and the WHD β-hairpin loop of the adjacent subunit. These interactions not only stabilize the active conformation but also facilitate an energetically favorable activation process (42).

Certain amphipathic molecules, including the chemotherapeutic agents, doxorubicin and paclitaxel, are capable of circumventing the canonical activation pathways and directly induce NLRP3 phase separation, thereby promoting inflammasome activation. This mechanism is believed to be intimately linked to changes in the solubility of protein regions, including the FISNA domains (41).

## 3 Signaling pathways of NLRP3 inflammasome activation

The NLRP3 inflammasome is a cytosolic multiprotein complex of the innate immune system that belongs to the NLR family. It functions as a critical sensor, detecting both exogenous pathogen invasion and endogenous cellular damage (39). As an intracellular danger-sensing platform, the NLRP3 inflammasome is activated in response to a wide range of PAMPs and DAMPs (43). The primary function of this pathway is to assemble a multiprotein complex that promotes the maturation and secretion of pro-inflammatory cytokines while initiating pyroptosis (44). Depending on the nature of the stimuli and the cellular context, NLRP3 inflammasome activation is classified into canonical and non-canonical pathways.

### 3.1 Canonical activation pathway

The canonical pathway generally necessitates two sequential signals: a priming signal and an activation signal. The priming signal is initiated when PRRs, such as TLRs, recognize PAMPs (e.g., lipopolysaccharide, LPS) or inflammatory cytokines (e.g., TNF-α). This signal activates the NF-κB pathway, resulting in the upregulation of NLRP3, pro-IL-1β, and pro-IL-18 expression. Consequently, cells enter a “primed” state, thereby enabling them to respond to subsequent activation signals (45).

**Figure 3:**
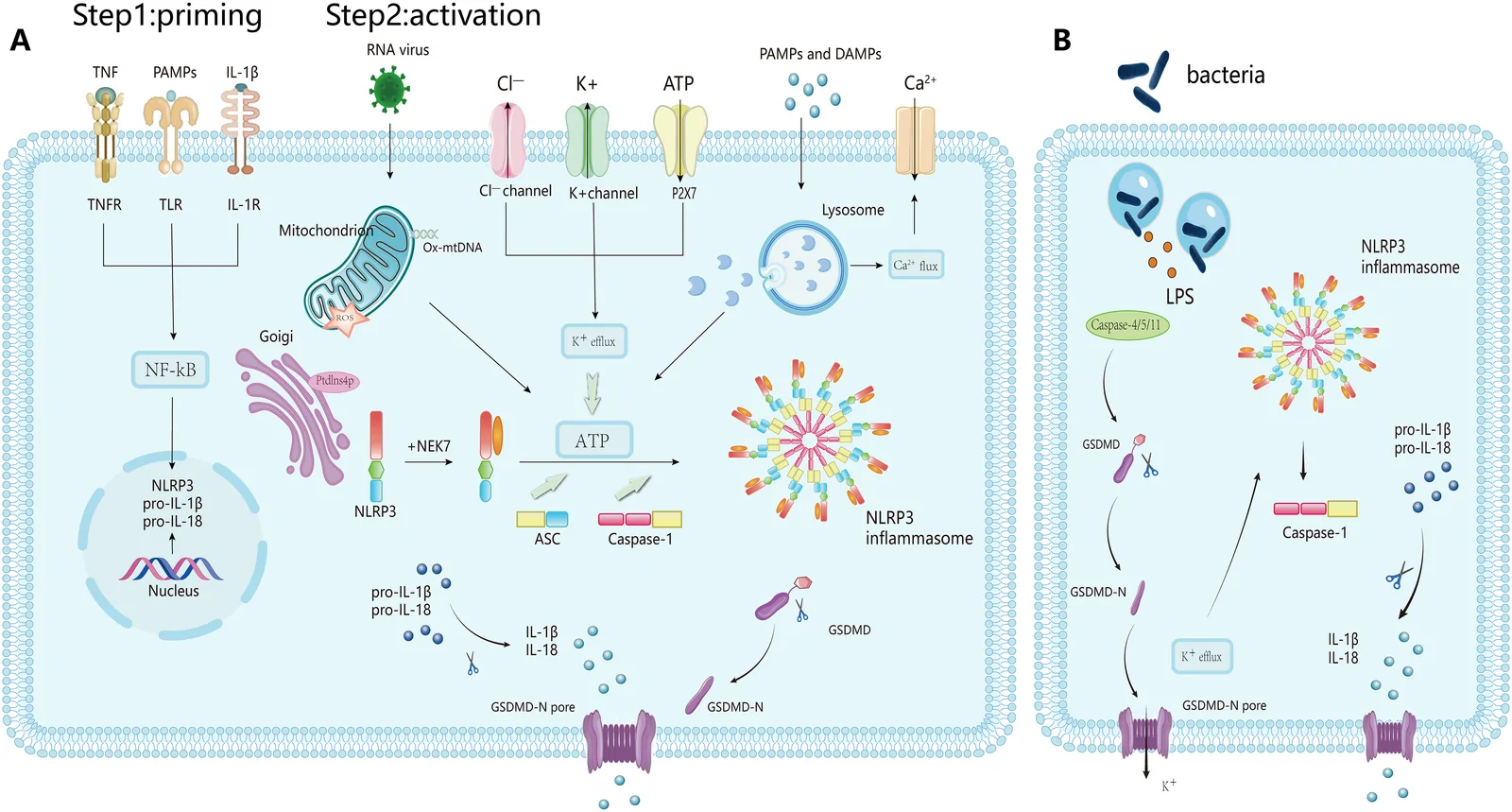
Molecular mechanisms of NLRP3 inflammasome activation. **(A)** Canonical NLRP3 activation pathway. This pathway consists of two steps: priming and activation. Priming phase induces NF-κB–dependent upregulation of NLRP3, pro–IL-1β, and pro–IL-18 via cytokine or pattern recognition receptor signaling. Activation is triggered by diverse PAMPs/DAMPs, including RNA viruses, ATP, and crystalline substances, which induce K^+^ efflux, mitochondrial dysfunction, ROS generation, mtDNA release, lysosomal damage, and Golgi dispersion. Dispersed Golgi membranes recruit NLRP3 via electrostatic interactions with phosphatidylinositol-4-phosphate (PtdIns4P). NLRP3 subsequently binds NEK7, recruits ASC and caspase-1, and forms the active inflammasome. Caspase-1 cleaves pro–IL-1β, pro–IL-18, and GSDMD to generate mature IL-1β, IL-18, and GSDMD-NT, thereby promoting cytokine release and pyroptosis. **(B)** Noncanonical NLRP3 activation pathway. Cytosolic LPS from Gram-negative bacteria directly activates caspase-4/5/11, resulting in GSDMD cleavage and pyroptosis. The associated K^+^ efflux subsequently triggers activation of the canonical NLRP3 inflammasome pathway.

The activation signal is initiated by structurally diverse danger factors, which subsequently lead to oligomeric assembly of NLRP3. The factors contributing to this process include the presence of extracellular ATP, crystalline substances (such as monosodium urate and cholesterol), pathogen-derived toxins, and organelle damage. Despite their diversity, these stimuli converge on several shared cellular events (15), primarily including ion fluxes, which involves K+ efflux, Ca2+ influx, and Cl− efflux (46); mitochondrial dysfunction, characterized by mitochondrial damage, mitochondrial ROS (mtROS) generation, and the release of oxidized mitochondrial DNA into the cytosol (47, 48); lysosomal damage, in which phagocytosis of crystals or granular material causes lysosomal membrane rupture and the release of lysosomal contents (49); and Golgi apparatus remodeling, during which dispersion of the trans-Golgi network generates phosphatidylinositol-4-phosphate (PtdIns4P), which recruits NLRP3 through electrostatic interactions and provides a platform for its aggregation and activation (50, 51). Upon stimulation by these signals, primed NLRP3 undergoes activation to assemble the inflammasome complex, which ultimately triggers pyroptosis (52) (**Figure 3A**).

### 3.2 Non-canonical activation pathway

In contrast to the canonical pathway, the non-canonical activation pathway does not require a priming step and is directly initiated by LPS in the cytoplasm. In humans, this pathway is mediated by caspase-4 and caspase-5, whereas in mice it is mediated by the orthologous caspase-11. During Gram-negative bacterial infection, bacterial LPS enters the host cell cytosol through mechanisms such as bacterial secretion systems, where it is directly recognized and bound by caspase-4/5/11 (53). This interaction promotes oligomerization and autoactivation of these inflammatory caspases, which subsequently cleave GSDMD to initiate pyroptosis (26).

The alteration of cell membrane permeability and the imbalance of ion homeostasis (K^+^ efflux) induced by GSDMD-mediated pyroptosis provide one of the key activation signals for the canonical NLRP3 inflammasome pathway (26). Consequently, the non-canonical pathway indirectly facilitates the canonical assembly of the NLRP3 inflammasome through the initiation of pyroptosis (54). These cytokines play pivotal roles in recruiting immune cells and amplifying both local and systemic inflammation. The NLRP3 inflammasome plays a significant role in metabolic diseases, autoinflammatory diseases, cardiovascular diseases, and autoimmune diseases (55–58) (**Figure 3B**).

### 3.3 Specific upstream triggers of NLRP3 inflammasome activation in LN

Although K^+^ efflux, mitochondrial ROS production, and lysosomal rupture are well-established mechanisms of NLRP3 inflammasome activation, these canonical pathways are triggered and amplified by several LN-specific upstream factors. The identification of these disease-specific triggers is essential for elucidating the association between NLRP3 inflammasome activation and LN pathogenesis.

In the kidneys of patients with LN, the deposition of ICs formed by dsDNA, anti-dsDNA antibodies, and complement components along the glomerular basement membrane represents an early and critical event in disease pathogenesis. The deposition of these ICs not only compromise glomerular filtration function but also to act as potent bioactive triggers, inducing innate immune responses (11). Endocytosis of ICs by renal resident innate cells (e.g., podocytes, mesangial cells) or kidney-infiltrating immune cells exposes their nucleic acid components (59). These endogenous nucleic acid fragments are subsequently recognized by TLR7 and TLR9, leading to activation of the NF-κB signaling pathway (60, 61), and priming of the NLRP3 inflammasome (62).

Immune complex deposition also activates the classical complement pathway, leading to the generation of large quantities of anaphylatoxins C3a and C5a (11). The binding of C5a to C5a receptor 1 (C5aR1), which is expressed on the neutrophil surface, has been demonstrated to promote the recruitment and infiltration of neutrophils into the renal glomeruli and interstitium. This inflammatory infiltration acts synergistically with TLR signaling (17, 61), providing a sufficient cellular basis and upstream signaling input for NLRP3 inflammasome activation (62). Upon stimulation by specific triggers, infiltrated renal neutrophils undergo neutrophil extracellular trap (NET) formation (NETosis), releasing reticular structures composed of histones, double-stranded DNA, myeloperoxidase (MPO), and other components (61). These structures not only function as potent TLR agonists but also sustain NF-κB-mediated priming, thereby further promoting NLRP3 inflammasome activation (62, 63).

A distinctive hallmark of SLE is the presence of a “type I interferon signature”, which is characterized by persistently elevated circulating levels of type I interferon (IFN-I) (64). IFN-I signal can induce the activation of the NLRP3 inflammasome in the context of LN. IFN-α synergizes with ICs to induce high expression of LY6E, which promotes excessive generation of mtROS, oxidative damage to mtDNA, and its cytoplasmic release. This cascade ultimately activates the cGAS/STING and NLRP3 signaling pathways, which exacerbate renal inflammatory infiltration (65). In glomerular podocytes, interferon regulatory factor 2 (IRF2) promotes inflammasome assembly and induces podocyte pyroptosis via the dual upregulation of NLRP3 expression and intracellular Ca²^+^ levels, which consequently disrupts the renal filtration barrier (66). Furthermore, existing studies have demonstrated that SIRT1 suppresses NLRP3 inflammasome activation by regulating the ROS/TRPM2/Ca^2+^ signaling pathway, consequently delaying the progression of LN. This finding further confirms the core role of the oxidative stress-mitochondrial damage-NLRP3 activation axis in the pathogenesis of LN (67).

Collectively, NLRP3 inflammasome activation in LN is driven by an integrated upstream signaling network rather than by a single pathway. The network under consideration comprises immune complex–TLR signaling, the complement–neutrophil axis, NET formation, IFN-I, and oxidative stress–mitochondrial damage (68). These signals are interconnected and undergo synergistic amplification, ultimately resulting in podocyte loss, proteinuria, and renal failure (69).

## 4 Role of the NLRP3 inflammasome in LN

The NLRP3 inflammasome plays a pivotal role in the pathogenesis of LN. It facilitates the release of pro-inflammatory cytokines, initiates pyroptosis, and extensively interacts with other signaling pathways to promote inflammatory injury, immune cell infiltration, and tissue fibrosis in the kidney (70). Aberrant activation of the NLRP3 inflammasome contribute to inflammatory responses and renal damage by influencing several key renal cell types (62). Specifically, the NLRP3 inflammasome is activated not only in conventional immune cells but also in kidney-resident cells, forming a complex cellular network that collectively promotes the initiation and progression of LN (63).

### 4.1 Role of the NLRP3 inflammasome in kidney-resident cell injury

The NLRP3 inflammasome, a pivotal sensor of the innate immune system, has been demonstrated to play a critical role in the pathological processes of various kidney diseases. Its expression is widely documented in multiple kidney-resident cell types (71). Upon stimulation of these cells, the NLRP3 inflammasome becomes activated, triggering robust inflammatory responses and pyroptosis that directly cause cellular injury and dysfunction. The persistent activation of these pathways contributes to renal fibrosis and progressive loss of kidney function (72).

#### 4.1.1 NLRP3 in podocyte injury

Podocyte injury is a critical pathological event leading to proteinuria in LN, and the NLRP3 inflammasome plays a direct pathogenic role in this process. The presence of an activated NLRP3 inflammasome has been identified in podocytes from both patients with LN and mouse models (73).

NLRP3 activation contribute to podocyte injury through several mechanisms:

Induction of pyroptosis: This is the most direct mechanism by which NLRP3 causes podocyte injury. Studies have shown that transcription factors including IRF2 and C/EBPβ induce podocyte pyroptosis by enhancing the expression of NLRP3 inflammasome components and modulating intracellular Ca^2+^ release (65, 74). The inhibition of the caspase-1/GSDMD signaling pathway effectively attenuate podocyte pyroptosis and ameliorate renal injury (75).

**Figure 4:**
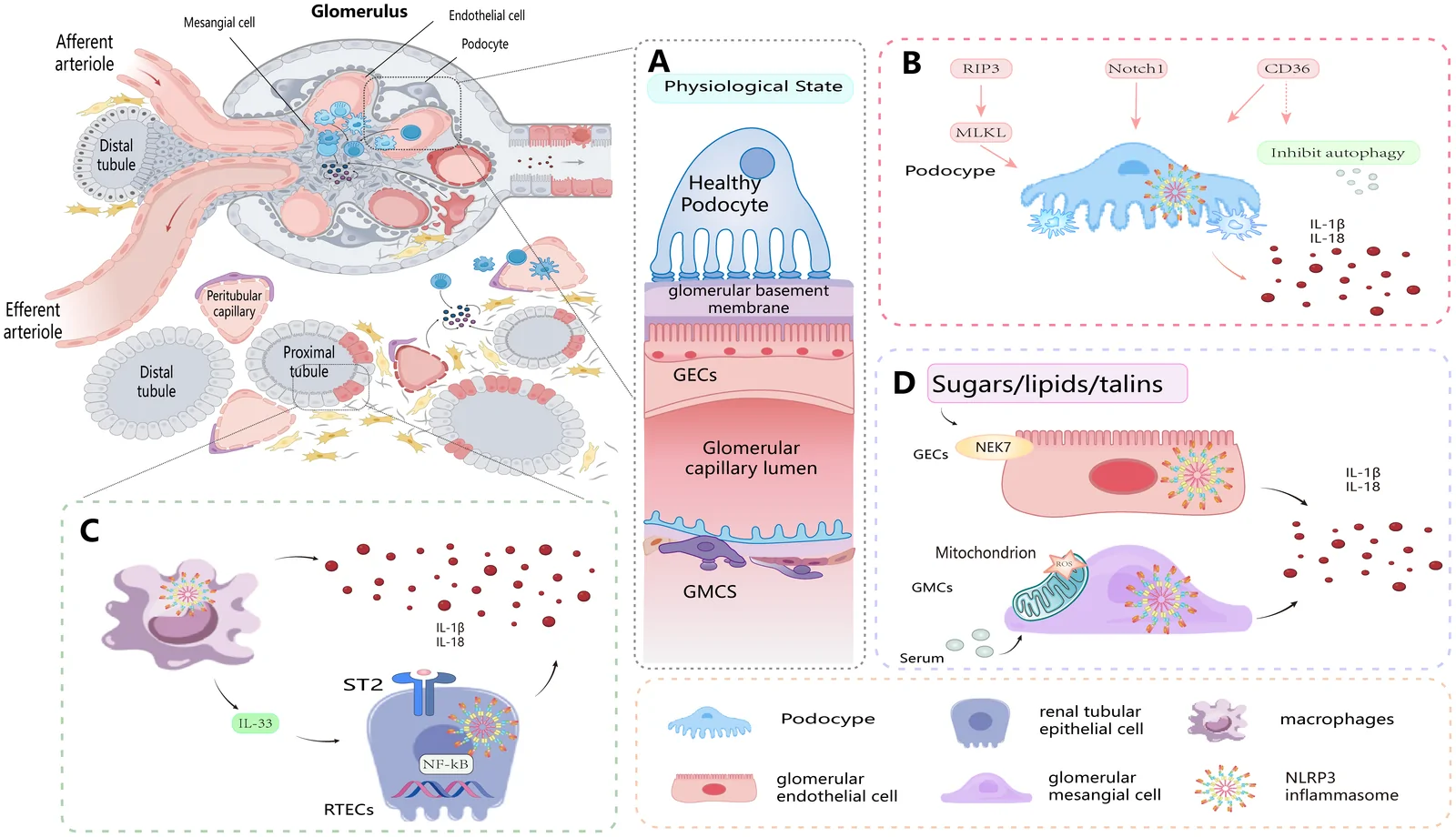
Roles of the NLRP3 inflammasome in renal innate cell injury during LN. **(A)** Schematic of podocytes, the glomerular basement membrane, glomerular endothelial cells, and mesangial cells under physiological conditions. **(B)** Role of the NLRP3 inflammasome in podocyte injury during LN. **(C)** Crosstalk between renal tubular epithelial cells and kidney-resident macrophages. **(D)** Roles of the NLRP3 inflammasome in glomerular endothelial cells and mesangial cell injury during LN.

Activation of the RIP3 pathway: RIP3-mediated necroptosis has been observed in podocytes from renal biopsy specimens of patients with grade IV LN and in the kidneys of lupus-prone NZM2328 and MRL/LPR mice. RIP3 activation has been observed to be concomitant with the activation of its downstream necroptosis effector MLKL and that of the NLRP3 inflammasome effector caspase-1. In NZM2328 mice, RIP3 activation becomes increasingly prominent in podocytes as LN progresses, suggesting that this pathway plays an important role in disease pathogenesis. In addition to initiating necroptosis, RIP3 promotes NLRP3 inflammasome activation. Consequently, in LN podocytes, RIP3 is activated in conjunction with the activation of its downstream molecules MLKL and caspase-1 (76) (**Figures 4A, B**).

CD36 signaling pathway: CD36 has been identified as a contributing factor to podocyte injury in various forms of glomerular disease and as an important candidate molecule in LN. CD36 is highly expressed on the surface of podocytes in LN, and promotes NLRP3 inflammasome activation while simultaneously suppressing autophagy, thereby exacerbating podocyte injury. In contrast, CD36 knockdown reduces NLRP3 expression, enhance autophagy, and alleviate injury (69) (**Figures 4A, B**).

Notch1 pathway: The Notch1 signaling pathway plays a pivotal role in the process of podocyte injury. In LN, aberrant activation of Notch1 promotes NLRP3 inflammasome activation. Treatment of injured podocytes with DAPT, a specific Notch1 inhibitor, significantly reduce the expression of NICD1 and NLRP3 while increasing the expression of the podocyte marker WT1 (a protein critical for podocyte health and differentiation), thereby alleviating podocyte injury. In animal models, the inhibition of the Notch1 pathway also reduce renal inflammation, alleviate podocyte injury, and decrease the levels of NLRP3 in podocytes. These findings suggest that the Notch1 pathway activates the NLRP3 inflammasome, leading to inflammatory injury and dysfunction of podocytes (77) (**Figures 4A, B**).

#### 4.1.2 NLRP3 in mesangial cell and glomerular endothelial cell injury

GMCs have historically been considered to function as structural support cells. However, under pathological conditions such as LN, mesangial cells undergo activation and become actively involved in the inflammatory response (11). GMCs have been shown to produce proinflammatory cytokines and extracellular matrix in LN, thereby contributing to the progression of glomerular fibrosis (78). Serum from LN patients has been shown to induce oxidative stress, apoptosis, and mitochondrial damage in human glomerular mesangial cells while concurrently activating the NLRP3 signaling pathway (79). These findings suggest that circulating pathogenic factors, including autoantibodies and immune complexes, can directly target mesangial cells and promote the assembly and activation of the NLRP3 inflammasome (**Figures 4A, D**).

Glomerular endothelial cells (GECs) have been shown to play an active role in the modulation of the immune system within the context of LN. Autophagy functions as a significant cytoprotective mechanism that exerts a negative regulatory effect on the NLRP3 inflammasome in GECs. In LN models, the suppression of mitophagy in GECs by IL-1β derived from steroid-resistant macrophages has been observed to activate the NLRP3 inflammasome, leading to exacerbated renal inflammation and tissue damage (80). In addition, indoxyl sulfate has been reported to activate the NLRP3 inflammasome in endothelial cells via NEK7 upregulation, thereby triggering inflammation and apoptosis associated with cardiovascular complications of end-stage renal disease (81). However, these findings have primarily been reported in models of metabolic nephropathy and end-stage renal disease, and direct evidence supporting a similar mechanism in LN remains limited and requires further investigation (**Figures 4A, D**).

#### 4.1.3 NLRP3 in renal tubular epithelial cells

RTECs are no longer regarded as passive victims but are recognized as core participants that actively engage in inflammatory and immune responses (11). Overactivation of the NLRP3 induces the secretion of pro-inflammatory factors and to recruit a substantial number of immune cells to infiltrate the renal interstitium. The release of cytokines by these infiltrating immune cells further exacerbate renal parenchymal injury. Furthermore, the expression level of NLRP3 in renal tubules is significantly correlated with the pathological activity of LN and the degree of renal function damage (82).

During the process of pyroptosis, the release of mature inflammatory mediators directly damages tubular epithelial cells, further enhances immune cell infiltration, and amplifies local inflammatory responses. These pathological processes have been successfully reproduced *in vitro* by stimulating human proximal renal tubular epithelial (HK-2) cells with LPS and ATP. The pharmacological inhibition of the NLRP3 signaling pathway effectively suppresses pyroptosis and inflammasome activation (83).

A close functional interplay exists between RTECs and kidney-resident macrophages. Activation of the NLRP3 inflammasome in macrophages directly upregulates the expression of IL-33. Subsequently, IL-33 acts on the ST2 receptor on the surface of RTECs to activate the downstream NF-κB pathway, thereby further promoting the inflammatory response of RTECs. This process establishes a positive feedback loop driven by the NLRP3/IL-33/ST2 axis, which exacerbates tubulointerstitial inflammation (84). Furthermore, autoantibodies (e.g., anti-dsDNA antibodies) from patients with LN have been observed to directly target RTECs. Studies have confirmed that anti-dsDNA antibodies have the capacity to induce apoptosis in mouse RTECs and to activate the NF-κB/NLRP3/caspase-1/IL-1β/IL-18 signaling axis (85) (**Figure 4C**).

The available evidence regarding NLRP3 activation in podocytes, mesangial cells, glomerular endothelial cells, and RTECs collectively supports several important conclusions. First, NLRP3 is widely activated across multiple kidney-resident cell types. Secondly, aberrant crosstalk between macrophages and RTECs provides direct evidence that NLRP3-mediated signaling facilitates communication between infiltrating immune cells and kidney-resident cells in LN. Thirdly, a multitude of pathogenic pathways, comprising the RIP3, CD36, and Notch1 pathways, intersect at the level of NLRP3 via discrete upstream mechanisms, thereby establishing NLRP3 as a pivotal signaling nexus that integrates diverse pathogenic stimuli. Fourthly, NLRP3-mediated podocyte pyroptosis disrupts the glomerular filtration barrier and induce proteinuria. The compromised filtration barrier permits increased exposure of the renal tubules to pathogenic factors, such as autoantibodies and immune complexes. This, in turn, exacerbates NLRP3-dependent injury in RTECs. Subsequent to injury, RTECs secrete pro-inflammatory mediators that recruit additional immune cells, thereby further amplifying renal inflammation. However, the current body of evidence remains inadequate to substantiate direct NLRP3-mediated signaling between podocytes and mesangial cells or between podocytes and RTECs. While all of these kidney-resident cell types express components of the NLRP3 inflammasome in LN, the complete intercellular signaling network mediated by NLRP3 and the directionality of these interactions remain to be fully elucidated.

### 4.2 Activation and role of the NLRP3 inflammasome in key immune cells

In the kidneys of patients with LN, macrophage polarization and heterogeneity, NETosis, T-cell exhaustion, and dysregulated intercellular communication collectively contribute to the immune-mediated pathological injury characteristic of the disease. The key molecules of the NLRP3 inflammasome activation pathway are highly expressed not only in the renal innate cells but also in the infiltrating immune cells (17). Several immune cell populations serve as major sites of NLRP3 inflammasome activation. This activation is tightly regulated by multiple upstream signaling pathways and ultimately promotes the maturation and release of pro-inflammatory cytokines, thereby exacerbating renal inflammation and disease progression (65, 86–89).

#### 4.2.1 Role of the NLRP3 inflammasome in renal macrophages

Macrophages represent a critical category of infiltrating immune cells that play a pivotal role in the development of LN. During the active phase of the disease, the kidneys are predominantly infiltrated by pro-inflammatory M1 macrophages. These macrophages promote glomerular and tubulointerstitial injury through the release of large amounts of inflammatory cytokines (e.g., TNF-α, IL-1β) and ROS. Conversely, anti-inflammatory M2 macrophages become enriched during disease remission, where they contribute to tissue repair and inflammation resolution (90, 91). The NLRP3 inflammasome functions as a pivotal molecular switch that instigates macrophage dysfunction. Numerous studies have demonstrated that the NLRP3 inflammasome is aberrantly activated in renal macrophages of patients with LN and in animal models. The NLRP3 inflammasome directly exacerbates renal injury and disease activity through the release of potent inflammatory factors (11, 17, 71, 86). The expression levels of NLRP3, ASC, caspase-1, IL-1β, and IL-18 are significantly elevated in the kidneys of patients with LN, with particularly high expression levels observed. The expression of these molecules is positively correlated with the SLEDAI and renal histopathological activity. This finding indicates that NLRP3 activation closely reflects disease severity (17).

The activation of the NLRP3 inflammasome in macrophages is subject to strict regulation by multiple upstream signaling pathways:

The S1P/S1PR1 signaling axis: Research has demonstrated that sphingosine-1-phosphate (S1P) levels are elevated in murine models of lupus, resulting in increased expression of S1PR1 on macrophages. Activation of the S1P/S1PR1 axis promotes the polarization of macrophages toward the pro-inflammatory M1 phenotype by enhancing NLRP3 inflammasome activation, thereby exacerbating renal inflammation (86).

NF-κB signaling pathway: NF-κB activation is indispensable for the transcriptional upregulation of NLRP3 and pro-IL-1β. A multitude of studies have shown that the inhibition of NF-κB signaling effectively suppresses NLRP3 inflammasome activation in macrophages and alleviates renal injury in LN (89, 92, 93).

ROS and Ca^2+^ signaling: ROS and Ca^2+^ influx are considered to be classic “activation signals” for the NLRP3 inflammasome in macrophages. SIRT1 has been shown to inhibit ROS production and thereby regulate TRPM2 channel-mediated Ca^2+^ influx, consequently inhibiting NLRP3 inflammasome assembly and activation (67). Furthermore, the mTOR pathway has been found to modulate NLRP3 expression and activation by regulating ROS levels (94).

The activation of the NLRP3 inflammasome in macrophages has been shown to play a pivotal role in the pathogenesis of renal injury, which is characterized by the release of inflammatory cytokines, the promotion of pyroptosis, the mediation of aberrant intercellular crosstalk, and the modulation of adaptive immune responses (71, 84, 93, 95).

#### 4.2.2 Role of the NLRP3 inflammasome in renal neutrophils

Neutrophils represent a pivotal immune cell type implicated in the pathogenesis of LN, with substantial accumulation observed within the glomeruli (96). Increasing evidence indicates that the activation of the NLRP3 inflammasome not only directly promotes local renal inflammation but also influences the progression and severity of LN through the regulation of neutrophil functions. IL-1β and IL-18 released during pyroptosis function as potent chemoattractants and activators of neutrophils, thereby promoting the adhesion and infiltration of circulating neutrophils into renal tissue (97–99).

Following stimulation by specific stimuli, recruited and activated neutrophils undergo a process known as NETosis, during which reticular structures composed of chromatin fibers, histones, and granule proteins, including myeloperoxidase (MPO) and neutrophil elastase (NE), are released. These NETs are recognized by macrophages, dendritic cells, and kidney-resident cells, thereby activating the TLR/NF-κB signaling pathway (priming signal) and the NLRP3 inflammasome itself (activation signal), leading to increased production of IL-1β and IL-18. Consequently, a positive feedback inflammatory loop is established: NLRP3 activation leads to neutrophil recruitment, NET release, and further activation of NLRP3, thereby sustaining and intensifying renal inflammation (100, 101). In addition, studies have identified NETs and citH3 as critical mediators that directly activate mesangial cells via TLR4. This activation has been shown to promote mesangial cell proliferation, increase the expression of pro-inflammatory cytokines and IFN-I-stimulated genes, and enhance collagen IV deposition. These findings establish the NET-mesangial cell axis as an important pathogenic mechanism driving LN progression (96).

#### 4.2.3 Role of the NLRP3 inflammasome in T cells

T cells fulfill a multitude of sophisticated functions in the pathogenesis of LN, contributing to immune cell infiltration, activation, and interactions with kidney-resident cells that eventually lead to renal injury and dysfunction. A notable infiltration of T cells, specifically CD4^+^ and CD8^+^ T cell subsets, is evident in renal tissues of patients with LN. The hypoxic microenvironment within inflamed renal tissue further aggravates disease progression. In such conditions, infiltrating CD4^+^ and CD8^+^ T cells express hypoxia-inducible factor-1 (HIF-1), which in turn reprograms cellular metabolism and promotes resistance to hypoxia-induced apoptosis. This adaptation facilitates the prolonged retention of T cells within injured renal tissues, thereby enabling their sustained activity (102, 103).

The impact of the NLRP3 inflammasome on T cell function can be conceptualized at two distinct levels. The initial mechanism under consideration is the well-characterized process involving IL-1β-mediated modulation of adaptive immunity. Upon NLRP3 inflammasome activation, the process of caspase-1 cleavage promotes the secretion of mature IL-1β and IL-18. These cytokines, particularly IL-1β, function as a critical link between innate and adaptive immunity. These cytokines have the capacity to modulate the differentiation of helper T cells with a high degree of effectiveness. Specifically, IL-1β has been shown to promote the polarization of CD4^+^ T cells into pro-inflammatory Th17 and Th1 subsets while concurrently inhibiting the immunosuppressive function of regulatory T (Treg) cells. This dual action of IL-1β contributes to its crucial role in T cell-mediated renal injury in LN. This mechanism has been confirmed in multiple autoimmune disease models (104). The second level is supported by emerging evidence suggesting that the NLRP3 inflammasome functions in renal tissue-resident memory T cells (TRMs) and directly contributes to tissue injury. Recent studies have identified a population of long-lived, highly activated CD8^+^CD69^+^CD103^+^ tissue-resident memory T cells in the kidneys of patients with LN. These cells manifest constitutive activation of the NLRP3 inflammasome, as evidenced by elevated expression of NLRP3, caspase-1, GSDMD, and IL-1β. This intrinsic inflammasome activity directly regulates the differentiation and effector function of TRM cells, thereby exacerbating renal pathological damage. Furthermore, IL-1β, produced endogenously by TRM cells, activates the NF-κB signaling pathway, which subsequently upregulates the expression of transforming growth factor-β receptor II (TGF-βRII) on T cells, thereby amplifying the inflammatory response (105). However, it should be noted that the majority of conclusions derived from this study are based on correlation analyses and pharmacological inhibition experiments, and lack direct causal evidence such as data from T cell-specific NLRP3 knockout models. Consequently, while these observations offer a novel research direction for elucidating the role of NLRP3 in LN, they should currently be regarded as preliminary and hypothesis-generating, pending validation by independent studies and more detailed mechanistic investigations.

### 4.3 An integrated model of NLRP3-driven progressive injury in LN

The pathogenesis of LN is a highly complex dynamic process involving interactions between systemic autoimmune responses and the local renal microenvironment (78). Instead of functioning independently, multiple cell types collectively form a bidirectionally regulated inflammatory network. Subsequent to the occurrence of inflammatory pyroptosis, glomerular podocytes, mesangial cells, and renal tubular epithelial cells release a substantial number of DAMPs. These DAMPs subsequently recruit macrophages, neutrophils, and tissue-resident T cells to infiltrate the renal parenchyma. Following the infiltration of immune cells, the secretion of pro-inflammatory factors, including IL-1β and IL-18, occurs. These factors serve to exacerbate injury to kidney-resident cells. This reciprocal interaction establishes a self-amplifying inflammatory loop that ultimately drives glomerular hypercellularity, crescent formation, tubular atrophy, and renal interstitial fibrosis (106, 107).

**Figure 5:**
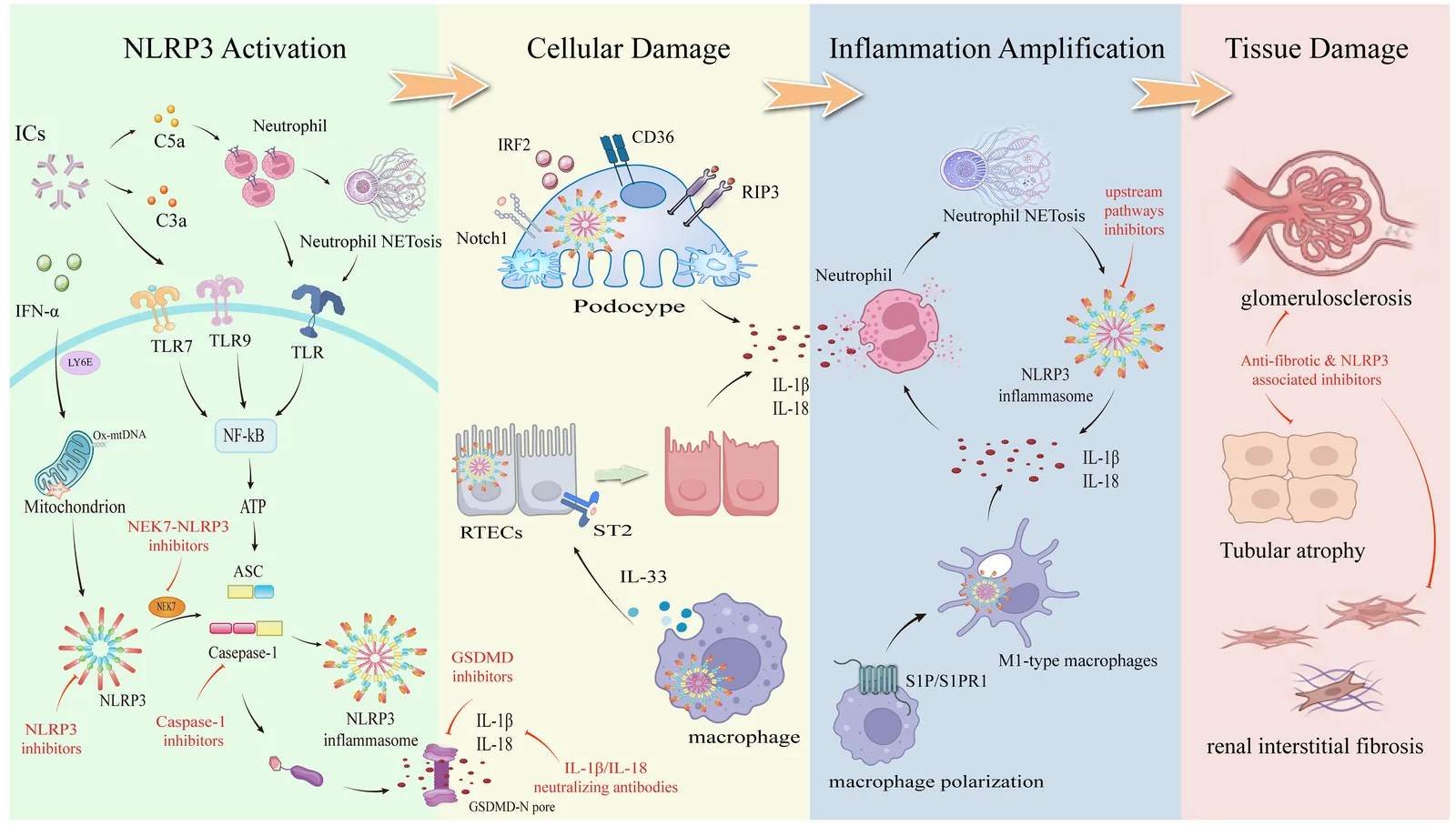
An integrated model of NLRP3-driven progressive kidney injury in lupus nephritis. The model depicts four interconnected phases from molecular activation to organ-level pathology. Phase 1: The activation of NLRP3 upon sensing LN-associated stimuli (immune complexes, dsDNA, complement activation, NETosis, IFN- I). Phase 2: Pyroptosis execution and initial injury in podocytes, renal tubular epithelial cells (RTECs), and macrophages. Phase 3: Immune cell recruitment and positive feedback loops, including neutrophil NETosis, macrophage M1 polarization. Phase 4: Chronic tissue remodeling and progressive fibrosis, culminating in glomerulosclerosis, tubular atrophy, and interstitial fibrosis. Therapeutic intervention strategies corresponding to each phase are highlighted in red: Phase 1 can target direct NLRP3 inhibitors, NEK7-NLRP3 interaction inhibitors and caspase-1 inhibitors; Phase 2 can target GSDMD inhibitors or anti-IL-1β/IL-18 neutralizing antibodies; Phase 3 can target upstream pathway activation inhibitors; Phase 4 requires combination anti-fibrotic strategies.

Integrating the findings from the preceding sections on the structural biology of NLRP3, its activation signaling pathways, and the pathogenic roles in renal cells, this section presents a unified model of disease progression that synthesizes these previously independent lines of evidence. The fundamental logic of this model is predicated on the premise that renal injury in LN is not independently driven by a single molecular event or cell type in isolation. Instead, it is the result of a hierarchical cascade that begins with conformational activation of the NLRP3 protein, progresses through pyroptosis execution and immune circuit amplification, and culminates in irreversible tissue remodeling (**Figure 5**). This framework integrates NLRP3 structural dynamics, inflammasome activation, the synergistic pathogenic effects of renal innate cells and infiltrating immune cells, and therapeutic intervention targets into a comprehensive spatiotemporal axis, thereby offering a conceptual foundation for understanding progressive renal injury in LN.

#### 4.3.1 Molecular sensing and conformational activation

The LN microenvironment is abundant in a variety of NLRP3-activating stimuli, including anti-dsDNA antibodies, TLR7/9 activation, complement activation, NETosis, IFN-I signaling and mitochondrial DNA release. These stimuli ultimately act on the NLRP3 protein.

In its resting state, NLRP3 adopts an ADP-bound autoinhibitory conformation. Upon ligand binding to the LRR domain, NEK7 disrupts the autoinhibitory structure via steric competition. Subsequent to this, the ADP-to-ATP exchange drives an approximate 85° rotation of the NACHT domain, which converts NLRP3 into active oligomers. These oligomers subsequently recruit ASC and pro-caspase-1 to complete inflammasome assembly (36, 38, 39). This conformational transition is the conserved molecular initiating event that underlies all subsequent cellular and tissue-level pathological processes in LN.

#### 4.3.2 Pyroptosis and initial cellular injury

The assembly of the NLRP3 inflammasome activates caspase-1, thereby initiating pyroptosis. In LN, pyroptosis manifests distinct spatiotemporal patterns that are specific to particular cell types. Podocyte pyroptosis, which is co-regulated by IRF2, C/EBPβ, and RIP3, directly induces foot process fusion and proteinuria (66, 74, 76). Pyroptosis of renal tubular epithelial cells induced by anti-dsDNA antibodies leads to tubular atrophy (11). In macrophages, the process of pyroptosis amplifies inflammation by transmitting damage signals to neighboring innate immune cells through paracrine mechanisms (71, 93). These findings suggest that pyroptosis is not merely a form of programmed cell death but also an active mechanism for damage signal transduction.

#### 4.3.3 Immune cell recruitment and the self-amplifying inflammatory network

The release of IL-1β, IL-18, and DAMPs by pyroptotic cell death and cell rupture initiates the recruitment and activation cascade of immune cells, establishing several interconnected positive feedback loops. These include the neutrophil–NETosis–NLRP3 reactivation loop (97–101), the S1P/S1PR1-mediated promotion of M1 macrophage polarization via the NLRP3 inflammasome (86), and the NLRP3/IL-33/ST2 signaling axis that drives the tubulointerstitial inflammatory loop (84). The core mechanism governing this stage is that NLRP3 acts not only as the initiator of the inflammatory loop but also as the target of the loop’s amplification effect.

#### 4.3.4 Tissue remodeling and progressive fibrosis

Persistent NLRP3-dependent and NLRP3-independent inflammatory signals drive irreversible tissue damage. Progressive loss of podocytes has been shown to induce glomerulosclerosis, while sustained injury and pyroptosis of RTECs result in tubular atrophy and interstitial fibroblast activation. IL-1β/TGF-β1 promotes myofibroblast transformation and stimulates the secretion of significant quantities of extracellular matrix, which can ultimately result in interstitial fibrosis (11, 17, 79, 108). This stage signifies the transition from reversible cellular damage to irreversible tissue destruction. Targeting NLRP3 at this stage rarely reverses established fibrosis, which further substantiates the importance of early intervention in LN treatment.

## 5 Current advances in NLRP3-targeted therapeutic strategies for LN

The central role of the NLRP3 inflammasome in the pathogenesis of LN has rendered it an attractive therapeutic target. Conventionally, LN immunosuppressants, which broadly suppress adaptive immune responses. In contrast, NLRP3-targeted therapies selectively interrupt the lupus-specific innate immune inflammatory cascade while also addressing the persistent injury of renal parenchymal cells. The therapeutic strategies that are currently employed to target the NLRP3 inflammasome can be categorized into four distinct classifications: direct inhibition of the NLRP3 inflammasome, blockade of upstream priming and activation signal axes, multitarget regulation by natural compounds, and gas therapy and other emerging physical interventions. This section offers a synopsis of the most recent advancements in NLRP3-targeted therapy for LN (**Table 1**).

**Table 1:** The application of therapeutic approach targeting NLRP3 in lupus nephritis treatment.

Classification	Drug/target	Mechanism of action	Key effects	Experimental stage	Ref.
Direct targeting of the NLRP3 inflammasome	MCC950/NACHT domain of NLRP3	Binds to the NACHT domain of NLRP3 to block inflammasome oligomerization and ATP hydrolysis, thereby inhibiting inflammasome activation; interrupts the positive feedback loop of IL-1β/NF-κB/TGFβRII	Downregulates caspase-1 and IL-1β; alleviates podocyte fusion, proteinuria, glomerular immune deposition and interstitial inflammation; suppresses the function of pathogenic tissue-resident memory (TRM) cells	Preclinical - *in vivo* animal experiments (NZM2328, MRL/lpr mice)	(73, 105, 109)
Targeting upstream transcriptional priming signals	Honokiol (HNK)/NF-κB	Inhibits IκBα phosphorylation to block NF-κB nuclear translocation; eliminates mitochondrial ROS to reduce upstream priming signals	Reduces renal NLRP3, activated caspase-1 and IL-1β; attenuates crescent formation and IgG/C3 deposition; suppresses Th1/Th17 cells and expands regulatory T cells (Tregs)	Preclinical - *in vivo* animal experiments (ASLN model)	(99)
	STAT1 knockout/STAT1	Blocks interferon-dependent transcriptional upregulation of NLRP3, pro-IL-1β and pro-IL-18, and reduces cellular priming sensitivity	Lowers renal expression of NLRP3, activated caspase-1, IL-1β and IL-18; alleviates glomerular hyperplasia and interstitial inflammation	Preclinical - *in vivo* animal experiments (MRL/lpr mice)	(112)
Targeting upstream metabolic and stress-derived activating signals	Piperine/AMPK	Activates AMPK to suppress NLRP3 activation and block tubular epithelial cell pyroptosis	Reduces mitochondrial ROS accumulation and inhibits NLRP3 inflammasome assembly	Preclinical - *in vivo* animal experiments/*in vitro* cellular experiments	(83)
	INK128, CB839/mTOR, glutamine metabolism	Dual inhibition of mTORC1/2 or blockade of glutamine metabolism to reduce mitochondrial ROS production	Suppresses NLRP3 activation and alleviates renal inflammatory injury	Preclinical - *in vivo* animal experiments/*in vitro* cellular experiments	(94, 115, 116)
	GSK872/RIP3 kinase	Disrupts the interaction between RIP3 and NLRP3, cutting off the crosstalk between necroptosis and inflammasome pathways	Reduces proteinuria and autoantibody levels, and markedly ameliorates renal pathological lesions	Preclinical - *in vivo* animal experiments (LN mice)	(76)
	LY6E/mitochondrial CMPK2	Inhibits the LY6E-CMPK2 axis to reduce mtROS release and oxidized mtDNA leakage, and blocks the cGAS/STING-NLRP3 cascade	Mitigates type I interferon-mediated intrinsic renal immune inflammation	Preclinical - mechanistic research/*in vitro* cellular validation	(65)
Targeting pathogenic signals from cell surface receptors	SSO, CD36 antagonistic peptide/CD36	Blocks endocytic uptake of lupus IgG to reduce ROS generation; restores autophagy-mediated NLRP3 degradation	Downregulates caspase-1 and IL-1β; relieves proteinuria and podocyte injury	Preclinical - *in vivo* animal experiments (MRL/lpr mice)	(69)
	DAPT/Notch1	Inhibits γ-secretase to block Notch1-NICD1 signaling and downregulate NLRP3 transcription; suppresses pro-inflammatory M2b macrophage polarization	Alleviates podocyte injury and glomerular immune deposition	Preclinical - *in vivo* animal experiments (MRL/lpr mice)	(77)
Multi-target natural compounds with synergistic regulatory effects	Melatonin, EGCG/SIRT1/Nrf2	Activates the Nrf2 antioxidant pathway to scavenge ROS and inhibit NF-κB transcriptional activity	Attenuates glomerular mesangial hyperplasia and oxidative damage	Preclinical - *in vivo* animal experiments (LN mice)	(117, 118)
	FGF21/Irgm1-dependent autophagy	Upregulates Irgm1 to mediate selective autophagic degradation of NLRP3	Promotes NLRP3 degradation and inhibits inflammasome activation	Preclinical - *in vivo* animal experiments/*in vitro* cellular experiments	(87)
	Ginsenoside M1/SIRT1-mediated autophagy	Upregulates SIRT1 to activate autophagy, which dually blocks NLRP3 priming and assembly; simultaneously modulates Th1/Th17 cells and Tregs	Suppresses excessive activation of innate and adaptive immunity and mitigates renal inflammation	Preclinical - *in vivo* animal experiments	(95)
	Ursolic acid (UA)/SUMOylation modification	Inhibits SUMO1-mediated NLRP3 SUMOylation to accelerate NLRP3 ubiquitin-dependent degradation, and blocks ASC speck formation and pyroptosis	Reduces NLRP3 stability and inhibits inflammasome assembly and cellular pyroptosis	Preclinical - *in vivo* animal experiments/*in vitro* cellular experiments	(119)
	Procyanidin B2/NLRP3 expression	Directly downregulates the expression of NLRP3, ASC and pro-caspase-1, with comparable efficacy to NLRP3 gene silencing	Potently suppresses the expression of all core inflammasome components	Preclinical - *in vitro* cellular experiments	(120)
Gaseous therapy and novel therapeutic agents	Xenon (Xe)/multi-pathway regulation	Upregulates HIF-1α/SIRT3 to restrain mitochondrial ROS burst; downregulates NF-κB; enhances autophagy to accelerate NLRP3 degradation	Reduces glomerular IgG/C3 deposition; inhibits immune cell infiltration; lowers proteinuria and anti-dsDNA antibody levels	Preclinical - *in vivo* animal experiments (ASLN, NZB/W mice)	(97)
	Tris DBA/ERK/JNK and autophagy	Inhibits ERK/JNK phosphorylation to block NLRP3 priming; upregulates Atg5/LC3B to induce autophagy and accelerate NLRP3 degradation	Reduces anti-dsDNA antibodies; ameliorates crescent lesions and proteinuria; expands Tregs and suppresses effector T/B cells	Preclinical - *in vivo* animal experiments (ASLN, NZB/W mice)	(121)

### 5.1 Direct inhibition of the NLRP3 inflammasome

MCC950 is a highly selective small-molecule inhibitor of NLRP3. It has been demonstrated that the compound binds to the NACHT domain of NLRP3, thereby impeding oligomeric assembly, inhibiting ATP hydrolysis, and inducing conformational inactivation of NLRP3 (109). This series of actions culminates in the suppression of NLRP3 activation. In the NZM2328 and MRL/lpr lupus mouse models, MCC950 has been observed to inhibit the NLRP3 pathway in both glomerular podocytes and renal CD8^+^CD69^+^CD103^+^ tissue-resident memory T cells, and to downregulate the expression of caspase-1 and IL-1β. Treatment with MCC950 attenuates podocyte foot process effacement, decrease proteinuria, reduce glomerular immune deposition and renal interstitial lymphocyte infiltration, disrupt the IL-1β/NF-κB/TGFβRII positive feedback loop, and suppress the proliferation and effector functions of pathogenic tissue-resident memory T cells (73, 105). However, at high doses, such accumulation may induce mitochondrial dysfunction or trigger unexpected off-target effects, leading to elevated liver enzyme levels and hepatocyte injury (110). A comparison of synthetic small-molecule inhibitors, exemplified by MCC950, with natural compounds reveals that the former exhibit enhanced specificity and augmented anti-inflammatory properties. However, their renal accumulation remains relatively limited, and their therapeutic efficacy has thus far been demonstrated mainly in preclinical animal models.

### 5.2 Targeting upstream activation signaling pathways

The activation of the NLRP3 inflammasome is governed by a signaling cascade that is characterized by a high degree of regulation and hierarchical structure. Consequently, upstream pathway modulators can be classified into three distinct categories based on their level of intervention.

#### 5.2.1 Targeting transcriptional priming signals

During the priming phase, transcription factors including NF-κB and STAT1 upregulate the expression of NLRP3, pro-IL-1β, and pro-IL-18. This results in the placement of cells in a “primed-sensitive” state. Stimuli such as lupus autoantibodies, immune complexes, and LPS activate the NF-κB and JAK/STAT signaling pathways via TLR/IFN receptors. This activation provides the essential molecular basis for subsequent inflammasome assembly.

Strategies targeting NF-κB: Honokiol (HNK) suppresses the NF-κB signaling pathway through two complementary mechanisms. Firstly, it inhibits IκBα phosphorylation, thereby preventing NF-κB nuclear translocation. Secondly, it scavenges mtROS, thereby attenuating upstream NF-κB activation. In the severe form of lupus nephritis (ASLN) model, HNK significantly downregulates renal expression levels of NLRP3, inactivates caspase-1 and suppresses IL-1β secretion, reduces glomerular crescent formation and IgG and C3 deposition, and concurrently suppresses the excessive activation of Th1/Th17 cells while promoting the expansion of Treg cells (99, 111).

Strategies targeting STAT1: STAT1 serves as the core transcription factor of interferon signaling and is a critical component in the study of interferon-based therapeutic interventions. The genetic knockout of STAT1 in MRL/lpr mice significantly reduce the expression of NLRP3 in the kidneys, leading to suppressed activation of caspase-1 and diminished secretion of IL-1β and IL-18. Concurrently, this process has been observed to attenuate glomerular proliferation and interstitial inflammation (112).

These strategies act at the most upstream step of NLRP3 activation and systemically reduce the “total output” of the inflammatory response. However, NF-κB and STAT1 are involved in a wide range of physiological processes, including immune development, cell survival, and metabolic regulation. Prolonged suppression of NF-κB and STAT1 has the potential to elevate the risk of infection and disrupt immune system balance. Furthermore, compensatory mechanisms within the NF-κB network, particularly activation of the non-canonical NF-κB pathway, may reduce therapeutic efficacy in humans compared with animal models. Moreover, existing therapeutic agents lack renal tropism, which complicates achieving effective intrarenal inhibition at safe doses (113, 114).

#### 5.2.2 Targeting metabolic and activation signaling pathways

Activation signals transduce a variety of cellular stress events, including mtROS generation, ion current dysregulation, and lysosomal damage, into direct triggering signals for NLRP3 oligomerization. The present intervention targeting this level includes AMPK/mTOR, RIP3, and LY6E, which are detailed as follows:

AMPK/mTOR metabolic pathway: The activation of AMPK inhibits NLRP3 inflammasome activation, while the overactivation of mTORC1/2 promotes NLRP3 expression via the mtROS pathway (83, 94, 115, 116). Piperine inhibits pyroptosis in renal tubular epithelial cells by activating AMPK (83). INK128, a dual inhibitor of mTORC1/2, and CB839, an inhibitor of glutamine metabolism, have been shown to inhibit NLRP3 activation by reducing mitochondrial ROS accumulation (94, 115).

RIP3 kinase: RIP3 functions as a core node, thereby establishing a connection between necroptosis and the NLRP3 inflammasome. Lupus autoantibodies induce RIP3 phosphorylation in podocytes, thereby activating both injury pathways concurrently. The RIP3 inhibitor GSK872 has been shown to impede the RIP3-NLRP3 interaction, decrease proteinuria and autoantibody levels, and substantially mitigate renal pathological damage (76).

LY6E: As an IFN-I inducible membrane protein, LY6E promotes the release of mtROS and oxidized mitochondrial DNA by upregulating mitochondrial CMPK2. This, in turn, has been observed to activate the cGAS/STING-NLRP3 signaling cascade. LY6E functions as an upstream bridging molecule that connects interferon signaling to NLRP3-mediated inflammation (65).

The targeting of these pathways offers enhanced specificity due to their proximity to the direct activation mechanism of NLRP3, thereby enabling comparatively higher intervention specificity. However, AMPK and mTOR function as core regulatory hubs of cellular energy metabolism, and prolonged modulation may result in metabolic disturbances, including hyperlipidemia and insulin resistance. RIP3 is involved in the maintenance of normal immune homeostasis, and its sustained inhibition may impair the host’s defense against infection. LY6E exerts a broad-spectrum antiviral function; therefore, its inhibition may increase the risk of viral infection. Furthermore, patients with LN exhibit high metabolic heterogeneity, and marked interspecies differences in metabolic pathways exist between humans and mice, while systemic administration fails to achieve sufficient drug concentration in the kidney. These three factors continue to impede the clinical translation of these therapeutic strategies.

#### 5.2.3 Targeting pathogenic signaling mediated by cell surface receptors

In LN, specific cell surface receptors have been shown to directly mediate pathogenic signals, ultimately leading to NLRP3 inflammasome activation.

CD36: CD36 demonstrates high expression in LN podocytes and promotes NLRP3 activation through dual mechanisms. It facilitates the uptake of lupus-derived immune complexes while concurrently enhancing intracellular ROS production, thereby promoting inflammasome assembly. Concurrently, CD36 exerts a suppressive effect on LC3-dependent autophagy, thereby inhibiting NLRP3 degradation. The CD36 antagonist sulfosuccinimidyl oleate (SSO) and the CD36 antagonist peptide have been shown to reduce caspase-1 and IL-1β, alleviate proteinuria, and attenuate podocyte injury in MRL/lpr mice (69).

Notch1: The activated intracellular fragment, NICD1, is highly expressed in LN podocytes and initiates inflammasome assembly through transcriptional up-regulation of NLRP3. The γ-secretase inhibitor DAPT blocks Notch1 signaling, reduce podocyte injury and glomerular immune deposition, and simultaneously inhibits the proinflammatory polarization of macrophage M2b (77).

These receptor-targeted strategies exhibit high specificity by selectively targeting LN-associated pathogenic pathways while concomitantly modulating lipotoxicity (CD36) or fibrosis-related signaling (Notch1). However, CD36 is imperative for the uptake of fatty acids and the clearance of apoptotic cells. A long-term CD36 blockade has the potential to induce lipid metabolism disorders. In addition, Notch1 is involved in various processes, including cell development, tissue repair, hematopoiesis, and conventional T cell differentiation. Therefore, sustained Notch1 inhibition may potentially disrupt normal tissue homeostasis. Notably, substantial interspecies disparities have been observed in renal lipid metabolism and Notch signaling between humans and mice. Systemic drug administration has been demonstrated to be ineffective in achieving precise targeting of glomerular podocytes. Furthermore, short-term anti-inflammatory interventions have been shown to be inadequate in reversing the development of chronic fibrosis during the progression of LN.

### 5.3 Multi-target regulation by natural compounds and phytochemicals

In contrast to chemically synthesized inhibitors, which generally target a single molecule, natural compounds have been shown to exert synergistic therapeutic effects by concurrently acting on multiple pathological processes, including innate immunity, adaptive immunity, oxidative stress regulation, and autophagy.

Nrf2 exerts regulatory effects on both antioxidant and anti-inflammatory processes. Melatonin and epigallocatechin gallate reduce mesangial proliferation and oxidative damage in LN mouse models by activating the SIRT1/Nrf2 signaling pathway, which facilitates ROS clearance and inhibits the transcriptional activity of NF-κB (117, 118).

Autophagy facilitates the degradation of NLRP3. Specifically, FGF21 mediate the selective autophagic degradation of NLRP3 by upregulating the expression of Irgm1 protein (87). Ginsenoside M1 induces autophagy activation via SIRT1 upregulation, thereby suppressing both the priming and assembly phases of NLRP3 inflammasome activation. Furthermore, ginsenoside M1 has been shown to impede the activation of Th1/Th17 cells, while concurrently promoting the differentiation of Treg cells. In addition to these effects, ginsenoside M1 has been observed to regulate both innate immunity and adaptive immunity (95).

The regulation of post-translational modifications is a subject of significant interest in the field of molecular biology. Post-translational modifications have been identified as critical determinants of NLRP3 protein stability. Ursolic acid (UA) inhibits SUMO1-mediated SUMOylation of NLRP3, accelerates the ubiquitination and degradation of NLRP3, and suppresses ASC speck formation and pyroptosis (119). Proanthocyanidin B2 directly downregulates the expression of NLRP3, ASC, and pro-caspase-1, exhibiting anti-inflammatory efficacy comparable to that achieved by NLRP3 gene silencing (120).

A comparison of natural compounds with synthetic inhibitors reveals that the former generally exhibit superior long-term safety profiles. Their multi-target characteristics enable simultaneous regulation of inflammation, oxidative stress, and immune dysregulation, rendering them particularly attractive for multifactorial diseases such as LN. However, due to their suboptimal oral bioavailability, inadequate renal targeting, and limited anti-inflammatory efficacy, natural compounds are currently suitable for adjuvant intervention only. Furthermore, metabolic differences between humans and mice, as well as metabolic heterogeneity among LN patients, pose significant obstacles to clinical translation.

### 5.4 Gas therapy and emerging therapeutic agents

As an inert medical anesthetic gas, Xenon (Xe) suppresses the NLRP3 cascade through multiple upstream signaling pathways. Specifically, it upregulates the HIF-1α/SIRT3 pathway to suppress mtROS generation, inhibit NF-κB transcriptional activity, and increase autophagy, thereby accelerating NLRP3 complex degradation. In ASLN and NZB/W lupus mice, Xe inhalation has been shown to reduce glomerular IgG/C3 deposition, inhibit macrophage/T cell infiltration, and reduce proteinuria and anti-dsDNA antibody levels (97). Xe exhibits a number of salient advantages, including antioxidant activity, suppression of innate immune responses, and modulation of autoantibody production. In addition, it produces fewer systemic adverse effects than conventional immunosuppressants. However, Xe necessitates airtight inhalation for drug delivery and is highly dependent on equipment. The study demonstrates limited feasibility for long-term outpatient administration, a lack of precise kidney-targeted drug delivery, and differences in human renal hypoxia-response pathways.

Tris DBA is a palladium-based small-molecule complex that exerts its biological effects via two distinct mechanisms: Tris DBA inhibits ERK/JNK phosphorylation and to block NLRP3 activation signaling. Furthermore, it has been observed to upregulate Atg5/LC3B, thereby inducing autophagy and accelerating the degradation of NLRP3 complexes. In the ASLN mouse models, Tris DBA reduces the production of anti-dsDNA antibodies, attenuates intrarenal immune cell infiltration, and ameliorates renal crescent formation and proteinuria. Furthermore, it has been demonstrated to expand the proportion of Tregs and to suppress the activation of effector T and B cells (121). However, palladium-based complexes are associated with the risk of metal accumulation and potential systemic toxicity. Moreover, the intraperitoneal administration of Tris DBA is the only method to achieve therapeutic efficacy. At this time, no oral formulation of Tris DBA is available. The primary impediments to the clinical translation of this compound are its inadequate renal targeting capacity and the absence of validated models for chronic renal fibrosis.

### 5.5 Comparative evaluation of therapeutic strategies

The four categories of NLRP3-targeted therapeutic strategies each possess distinct advantages and disadvantages with respect to specificity, safety, translatability, and stage-specific applicability. The direct NLRP3 inhibitors exhibits the highest degree of specificity and the strongest anti-inflammatory activity. However, their narrow safety window, potential hepatotoxicity, and limited renal accumulation currently restrict their use to preclinical studies. Upstream pathway modulators have the capacity to inhibit multiple inflammatory pathways; however, they frequently target molecules that play essential physiological roles. This characteristic increases the likelihood of adverse events related to off-target effects and immune-related adverse events. The majority of these targets are still currently limited to fundamental research, and the effectiveness observed in animal models has yet to be translated into clinical applications. Natural compounds have been demonstrated to exhibit an optimal safety profile and the advantage of multi-target synergistic regulation. However, their poor oral bioavailability and relatively weak anti-inflammatory efficacy limit their use mainly to adjunctive therapy. Gaseous mediators and metal complexes have shown significant efficacy in animal models. However, their dependence on specialized delivery systems, concerns regarding metal accumulation, and limited clinical feasibility represent major obstacles to their widespread application. In light of the aforementioned comparison, we hereby propose the following therapeutic framework. In the case of acute active LN, the administration of direct NLRP3 inhibitors has been proposed as a therapeutic strategy to expedite the management of inflammatory exacerbations. Secondly, the chronic maintenance phase necessitates the implementation of mild multi-target modulation through the utilization of natural compounds or pathway modulators. Thirdly, in cases that do not respond to other treatments, a strategy of low-dose multi-target inhibition (i.e., the simultaneous targeting of both initiation and activation signals) could be considered. This approach aims to achieve a synergistic enhancement of the treatment while reducing the toxicity of the individual agents.

In recent years, significant strides have been made in the development of NLRP3 inhibitors. A multitude of candidate inhibitors have entered various stages of clinical development for the treatment of other inflammatory diseases. Ruvonoflast (NodThera) is an orally bioavailable, brain-penetrant NLRP3 inhibitor. In a Phase 1b clinical trial that enrolled patients with elevated cardiovascular risk, 28 days of ruvonoflast treatment resulted in a significant reduction in high-sensitivity C-reactive protein (hsCRP) levels, with a reported decrease of 82.2%. Concurrently, the treatment led to substantial downregulation of inflammatory biomarkers, including IL-6 and fibrinogen (122). NT-0796, an ester-based prodrug, has been demonstrated to target NLRP3. Preclinical studies have confirmed its high efficiency in crossing the blood-brain barrier. In the domain of clinical research, NT-0796 has successfully concluded Phase 1b open-label trials, encompassing both healthy elderly participants and patients diagnosed with Parkinson’s disease (123). Dapansutrile (OLT1177^®^) represents a recently developed inhibitor of the NLRP3 pathway. A review of clinical trial data reveals that dapansutrile demonstrates a favorable safety and tolerability profile when administered through either topical gel or oral capsule. Furthermore, the efficacy of dapansutrile in alleviating joint pain has been shown to be satisfactory (124). Nevertheless, the fundamental concern pertains to the fact that none of the aforementioned clinical trials were specifically designed for patients with LN. Despite the encouraging therapeutic potential demonstrated by NLRP3-targeted therapies in preclinical LN models, there is currently a paucity of publicly disclosed clinical trial data characterizing the safety, pharmacokinetic properties, or clinical efficacy of NLRP3 inhibitors in LN patients.

## 6 Conclusions and future perspectives

This review underscores the pivotal function of the NLRP3 inflammasome in the pathogenesis of LN and provides a comprehensive overview of recent advancements in its development as a therapeutic target. In consideration of the extant data, the following conclusions can be derived: 1. The aberrant activation of the NLRP3 inflammasome constitutes a pivotal element in the genesis of renal inflammatory injury and disease progression in LN. 2. The NLRP3 inflammasome has been shown to establish a complex intercellular regulatory network by influencing various innate cells and infiltrating immune cells in the kidney. 3. A multitude of intervention strategies targeting the NLRP3 inflammasome and its upstream and downstream regulatory pathways have exhibited significant therapeutic potential in preclinical models. Despite the substantial advances that have been achieved in recent years in delineating the contribution of the NLRP3 inflammasome in the pathogenesis of LN, several core scientific questions remain unresolved: 1. The cell-type-specific functions of NLRP3 in heterogeneous renal cell populations remain to be fully elucidated. 2. The existing arsenal of NLRP3-targeting drugs exhibits notable deficiencies in terms of specificity and safety. 3. The establishment of a biomarker-based precision diagnosis and treatment system for LN is of the utmost importance.

In light of the aforementioned analysis, it is hereby proposed that the following research directions will shape the subsequent phase of NLRP3 research: (1) The utilization of single-cell and spatial omics technology is of particular significance in this field. Single-cell transcriptomics is a technique that can analyze the heterogeneity and activation status of different cell subsets in the kidney of patients with LN. Spatial transcriptomics and spatial proteomics further integrate molecular characteristics with tissue spatial structure to generate a spatial map of NLRP3-driven renal inflammation, thereby providing a foundation for precise staging and targeted intervention. (2) Cell-specific gene knockout model. The present studies primarily utilize global knockout mice, yet it remains challenging to discern the distinct functions of NLRP3 in various cell types. The generation of NLRP3 knockout mice that are specific to podocytes, renal tubular epithelial cells, and macrophages will facilitate the elucidation of the pathogenic contribution and signal transduction network of each cell type. (3) The process of PROTAC-mediated NLRP3 degradation. Conventional small molecule inhibitors, exemplified by MCC950, are characterized by limitations including off-target effects and liver toxicity. The advent of PROTAC technology has ushered in a novel paradigm for the precise regulation of the NLRP3 inflammasome by inducing ubiquitin-proteasome degradation of NLRP3 protein (125). Recent advancements have led to the successful development of NLRP3 PROTAC molecules, which are based on MCC950 analogues (126). (4) The application of artificial intelligence in the domain of drug discovery has emerged as a significant area of research interest. The employment of artificial intelligence (AI)-driven drug screening platforms has the potential to expedite the development of drugs that target the NLRP3 pathway. Machine learning methods have been successfully employed for virtual screening of NLRP3 inhibitors (127, 128), and AI generative models have been used to design novel derivatives with NLRP3 inhibitory activity. (5) The implementation of precision therapy, guided by biomarkers, and its subsequent clinical translation, is a subject of significant interest in the field. The following objectives are to be pursued: validate molecular biomarkers, including serum NLRP3 levels, ASC specks, IL-18, and GSDMD fragments, for patient stratification and treatment response prediction; conduct dedicated clinical trials for LN; explore combination therapeutic strategies of NLRP3-targeted drugs with standard-of-care therapies; and ultimately establish a clinically actionable precision medicine model.

The pathogenesis of LN has evolved from the traditional IC deposition paradigm to a complex network of interactions among innate immunity, adaptive immunity, and renal innate cells. The NLRP3 inflammasome serves as a crucial node in this network, and targeted intervention on it is expected to overcome the limitations of existing treatment options. Consequently, future therapeutic strategies for LN are expected to shift away from broad-spectrum immunosuppression toward more precise, multitargeted, and less toxic therapeutic approaches. With the ongoing development of mechanistic research, drug development, and precision medicine, therapies targeting the NLRP3 inflammasome are poised to transition from experimental studies into clinical practice, offering improved treatment options for patients with LN.
